# Code Status Documentation Availability and Accuracy Among Emergency Patients with End-stage Disease

**DOI:** 10.5811/westjem.2020.12.46801

**Published:** 2021-04-27

**Authors:** Evan Russell, Andrew K. Hall, Conor McKaigney, Craig Goldie, Ingrid Harle, Marco L.A. Sivilotti

**Affiliations:** * Queen’s University, Department of Emergency Medicine, Kingston, Ontario, Canada; † University of Calgary, Department of Emergency Medicine, Calgary, Alberta, Canada; ‡ Queen’s University, Department of Medicine, Division of Palliative Care, Kingston, Ontario, Canada; § Queen’s University, Department of Biomedical & Molecular Sciences, Kingston, Ontario, Canada

## Abstract

**Introduction:**

Some patients with end-stage disease who may neither want nor benefit from aggressive resuscitation receive such treatment if they cannot communicate in an emergency. Timely access to patients’ current resuscitation wishes, or “code status,” should be a key metric of electronic health records (EHR). We sought to determine what percentage of a cohort of patients with end-stage disease who present to the emergency department (ED) have accessible, code status documents, and for those who do, how quickly can this documentation be retrieved.

**Methods:**

In this cross-sectional study of ED patients with end-stage disease (eg, palliative care, metastatic malignancy, home oxygen, dialysis) conducted during purposefully sampled random accrual times we performed a standardized, timed review of available health records, including accompanying transfer documents. We also interviewed consenting patients and substitute decision makers to compare available code status documents to their current wishes.

**Results:**

Code status documentation was unavailable within 15 minutes of ED arrival in most cases (54/85, or 63%). Retrieval time was under five minutes in the rest, especially when “one click deep” in the EHR. When interviewed, 20/32 (63%) expressed “do not resuscitate” wishes, 10 of whom had no supporting documentation. Patients from assisted-living (odds ratio [OR] 6.7; 95% confidence interval [CI], 1.7–26) and long-term care facilities (OR 13; 95% CI, 2.5–65) were more likely to have a documented code status available compared to those living in the community.

**Conclusion:**

The majority of patients with end-stage disease, including half of those who would not wish resuscitation from cardiorespiratory arrest, did not have code status documents readily available upon arrival to our tertiary care ED. Patients living in the community with advanced disease may be at higher risk for unwanted resuscitative efforts should they present to hospital in extremis. While easily retrievable code status documentation within the EHR shows promise, its accuracy and validity remain important considerations.

## INTRODUCTION

Decisions surrounding the resuscitation of a dying patient are complex and time pressured, yet are often made by emergency healthcare providers with incomplete or inaccurate information. Attempts to resuscitate patients with end-stage disease are often futile, unnecessarily traumatic to the dying patient and family, and disturbing to healthcare providers.^1^–^3^ Despite this, many patients with end-stage disease receive invasive resuscitative interventions at the end of life despite their expressed or implied goals of care.^4^–^6^ Many of these patients who present to the emergency department (ED) at the end of life are often so ill that they are unable to communicate their goals of care, including their code status,^7^ or they lack the ability to make decisions about their care at the end of life.^8^ In the absence of a readily available substitute decision maker (SDM), patients’ wishes for resuscitation are best obtained through code status documentation. The complexity and time pressure surrounding the high-stakes decision whether to withhold resuscitative efforts has increased with the COVID-19 pandemic.^9^

While electronic health records (EHR) hold the promise of rapid information retrieval, in many settings this remains to be realized, especially for the more nuanced considerations surrounding a patient’s code status. We wondered how often patients with end-stage disease had code status documentation available at the time of ED arrival. We also sought to measure the delay to retrieval and the accuracy of this documentation compared to current resuscitation wishes.

## METHODS

### Design

This cross-sectional study occurred from mid-June to mid-August 2016 at a tertiary, academic acute-care hospital, functioning as the referral center for a catchment population of approximately 500,000, and with an ED census of 55,000 visits per year. Ethics approval was granted by the institutional research ethics board.

### Participant Recruitment

Inclusion criteria were developed by consensus among study authors. We enrolled consecutive patients who met at least one of five inclusion criteria: 1) palliative care consultation within the prior three months; 2) metastatic malignancy; 3) home oxygen use for chronic obstructive pulmonary disease or heart failure; 4) dialysis for chronic kidney disease; or 5) progressive neurodegenerative disease, including a documented diagnosis of dementia, regardless of severity. These criteria were meant to outline a patient cohort for whom, if possible, most emergency physicians would want to confirm their code statuses prior to proceeding with invasive resuscitation efforts. Subjects were not required to be critically ill to be enrolled in the study.

One author (ER) identified and recruited every eligible patient present in the ED during random convenience sampling, recruiting for 15–20 hours per week. This author (ER) was separate from the patient’s care team. Sampling times included dates from each day of the week, from 6 am – 2 am the following day. Over the course of the enrolment period, efforts were made to evenly distribute sampling times across days of the week and time of day. We identified eligible patients by scanning through the ED’s EHR to see whether they met the inclusion criteria. All patients who met inclusion criteria at the times when the recruiting author (ER) was present in the ED were enrolled using a standardized and structured protocol to try to retrieve code status documentation. The same author then approached the patient (or, if incapable, the SDM) to obtain consent for the interview portion of the study, after excluding those who were critically ill from the interview portion of the study. Outcome measurements which did not require patient interview were collected for every eligible patient, including those patients who were not interviewed.

**Population Health Research Capsule**

What do we already know about this issue?

*Patients with end-stage disease frequently present to the emergency department (ED) at the end of life, sometimes receiving unwanted resuscitation.*

What was the research question?

*What proportion of patients with end-stage disease presenting to the ED have accessible code status documents?*

What was the major finding of the study?

*Of 85 enrolled patients, 54 (63%) did not have any available code status documentation, either in paper or electronic form.*

How does this improve population health?

*This underscores the need to increase code status document rates and availability in the ED for patients in the community with end-stage disease.*

### Outcome Measures

The primary outcome measure was the retrieval of previously established code status documentation, either from accompanying documents or from the hospital EHR (QuadraMed CPR, Plano, TX). Secondary outcome measures included the time required to obtain the code status, the retrieval of our hospital’s “Patient’s Goals of Care Discussion Form,” and concordance between the documented code status as retrieved vs current wishes as expressed by the patient or SDM at the time of the interview.For the purposes of this study, code status was classified as either “full code” (ie, full resuscitative measures in case of cardiorespiratory arrest) or “DNR” (do not attempt resuscitation in that event). DNR was explicitly defined as direction to not perform chest compressions, defibrillation, and invasive ventilation. Code status falls under the broader umbrella of *goals of care*, which includes acceptability of other life-sustaining measures interventions, as well as medical and symptom management.^10^

### Data Collection

All enrolled patients had demographic information collected, as well as presenting complaint, and Canadian Triage Acuity Scale (CTAS) scores were recorded. The CTAS is a validated tool used in all Canadian EDs. When triaging patients, emergency nurses assign CTAS scores to patients, ranging from 1 – resuscitation, to 5 – non-urgent. The process of how a score is assigned is determined by patient complaint, specific modifiers (eg, patient age, vital signs), and nursing assessment.^11^

For the primary outcome measure, we performed a timed searched for existing documentation in the available medical records. We developed a search algorithm by consensus to mimic the steps an experienced emergency physician would use for a patient unable to communicate and in extremis. First, any accompanying paper documentation that had been brought with a patient was reviewed (termed “accompanying documentation”), such as transfer forms from a long-term care facility. Second, the patient’s EHR was searched in the following order: 1) selecting the single-click “Life Care Plans” icon on the patient’s homepage; 2) discharge summaries within the previous two years, 3) clinic reports within the previous two years, or 4) all Ministry of Health and long-term care forms (including past ambulance records with any prehospital advance directive form). The “Life Care Plans” icon was introduced into the EHR approximately two years prior to the study. It allows “one-click” access to scanned copies of both “Do Not Resuscitate Confirmation” ministry forms (Appendix A), as well as copies of the “Patient’s Goals of Care Discussion Form” (Appendix B). The latter, a standardized paper form at our hospital to capture goals of care discussions, was introduced approximately one year prior to the study with the expectation that it be completed routinely during the admission process. In addition to recording patients’ preferences on the scope of treatment they were willing to receive, the form also recorded a discussion of the patients’ understanding of their medical condition(s), their values, priorities, and expectations of treatment.

The two time intervals spent searching either through patients’ accompanying documentation or the EHR were each recorded separately. The timer was stopped as soon as the first documentation of code status had been located and read in sufficient detail to classify with confidence as “full code” or “DNR.” The search was terminated when all eligible records had been reviewed, or the elapsed time had surpassed 15 minutes of dedicated searching. This curfew was determined a priori as beyond the clinically relevant upper time limit during active resuscitation.

One author (ER) interviewed all patients who consented for interview. The partially scripted interview (Appendix C) included a question about the patient’s current code status. Patients (or their surrogate) were given the explicit options of “full code” or “DNR,” and each option was explained in lay language to the patient. If patients were uncertain of their present goals of care, their responses were deemed “full code.” Patients were also asked about their knowledge of laws governing resuscitation, attitudes about the importance of code status documentation availability in the ED, and about any past invasive resuscitation or intensive care unit (ICU) admission. We defined *invasive resuscitative measures* as any one of the following: chest compressions; non-elective intubation with mechanical ventilation; or defibrillation.

### Analysis

We selected a priori six variables to test for association with the primary outcome: place of residence; gender; age; number of hospitals admission in the past year; prior ICU admission; and prior invasive resuscitation. For all tests of statistical significance, *P* < 0.05 was considered significant.

## RESULTS

A total of 85 patients were enrolled in the study, of whom 32 (patient or SDM) were also interviewed (Table 1).

Only 31 (36%) enrolled patients had documented code status that could be retrieved at the time of ED presentation (Table 2).

**Figure 1:**
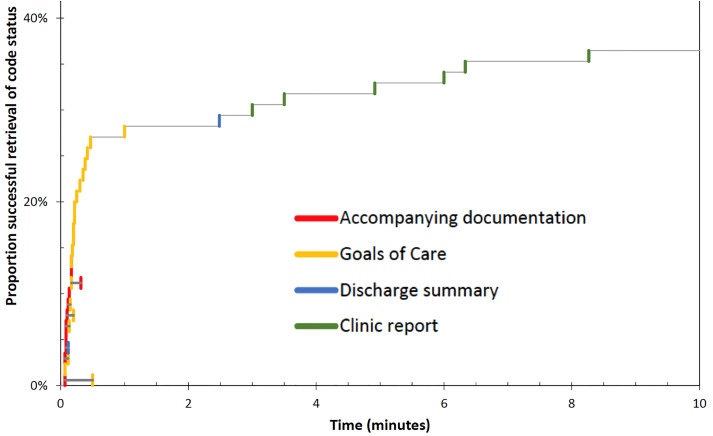
Time interval needed to obtain code status documentation from either accompanying paper documentation (Accompanying documentation), or the electronic health record (ie, “Goals of Care” icon, discharge summary, or clinic report) Note: When the search was terminated prior to the 15-minute mark because available records had been reviewed and did not contain code status documents, this time was not recorded.

When code status documentation was found, it was almost always available through the EHR (28 of 31 patients), and most commonly found using the single-click “Life Care Plans” icon on the EHR homepage. Of the 31 patients with code status documentation, 13 had accompanying documentation of the goals of care discussion. Not surprisingly, when available either via accompanying paper documentation or the “Life Care Plans” icon on the EHR, code status could be determined within one minute (Figure 1).

When code status documentation was retrieved via other means (eg, reviewing past discharge summaries or clinic reports), the mean (± standard deviation) time to retrieval was 4.33 ± 2.57 minutes. In the remaining 54 cases (63%), no code status documentation could be located, despite searching for up to 15 minutes. If found, documented code status agreed well but not perfectly with the current wishes of the patient (or SDM) (Table 3).

Of the 12 patients who self-identified as “full code” on interview, only three had documentation to support this. Of the 20 patients who self-identified as “DNR” on interview, only 10 had supporting documentation. There was one instance in which a SDM for a patient indicated that the patient would be full code despite code status documentation to the contrary, and another in which the accompanying paper documents indicated a patient’s code status as being “full code,” while both the EHR and patient interview identified the patient as DNR.

Of the variables studied (Table 4), only patient residence was associated with having available code status documents. Patients from assisted-living (odds ratio [OR] 6.7; 95% confidence interval [CI], 1.7–26) and long-term care facilities (OR 13; 95% CI, 2.5–65) were more likely to have a documented code status available compared to those living in the community.

Of the 32 patients or SDMs who consented to an interview, 14 (44%) reported being unaware that if a patient presents to the ED in extremis, resuscitation efforts would be initiated in the absence of a SDM or code status documentation saying otherwise. Twenty-seven (85%) of the patients interviewed thought that it was important for ED staff to know their code status. Twelve patients (37%) reported having an existing advance directive, and one had brought it to hospital.

## DISCUSSION

Most patients with end-stage disease, including half of those who would not wish resuscitation from cardiorespiratory arrest, did not have any code status documents readily available upon arrival to our ED. By focusing on code status availability in patients with end-stage disease processes at the time of ED presentation, we sought to explore the clinically important issue of how often such patients were at risk for unwanted or unnecessary resuscitative efforts if they were to arrive in extremis. Only one in three patients had code status documentation readily available on presentation to the ED, yet the majority of those interviewed agreed it was important for emergency physicians to have access to their documented code status. Reassuringly, when code status documentation was available, it was almost always consistent with the current wishes of the patient or SDM. Code status preferences are generally durable over time, especially for those wishing to restrict the invasiveness of their care.^12^

The availability and retrieval of code status at the time of ED presentation has not been well studied. In another single-center Canadian study, only 35% of 280 enrolled patients knew what an advance directive was, 19.3% had a documented advance directive, and 5.6% had brought it to the ED.^13^ Other studies of *admitted* patients found variable documentation rates, ranging from 0.53–10.3% for all admitted patients,^14^,^15^ rising to 30–36% for admitted patients with end-stage disease.^16^,^17^ For patients who are critically ill when admitted to hospital, or who reside in a long-term care facility, obtaining and documenting a code status is a well-established practice.^5^,^18^ While code status may not be documented on all discharge summaries, the immediate location and retrieval of a patient’s prior goals of care should be a priority for any health informatics system, comparable in many ways to allergy or prior violence alerts. While EHR systems continue to mature, our findings highlight the utility of designing an EHR with immediate access to a patient’s documented code status.

For those patients with code status documents, we demonstrated near immediate retrieval using either review of paper forms accompanying a transfer, or with a quick-access icon in the EHR. Retrieval times were much longer when reading through recent discharge summaries or clinic reports. Importantly, most of these latter sources failed to produce any information regarding code status even after a prolonged and concerted effort, leaving the residual uncertainty of whether the issue had ever been discussed. Limited access to palliative care patients’ full medical record is a major barrier to providing quality, patient-centered palliative care in the ED.^19^ Moving forward, healthcare systems should require greater information integration across clinical environments (ie, ED, family physician offices, outpatient clinics, inpatient services).^13^,^20^ There are several examples of rapid access, centralized code-status systems for frontline emergency healthcare providers in other jurisdictions that warrant exploration.^21^–^24^

Of all patients interviewed, the majority self-identified as “DNR,” despite many lacking documentation to support this assertion. These patients could have received unwanted resuscitations had they presented to hospital in extremis, absent a knowledgeable SDM. This unacceptably high number may be partially driven by only half of interviewed patients being aware that full resuscitative efforts could be initiated in the absence of documentation or an SDM to say otherwise.

More than one-third of recruited patients interviewed self-identified as “full code,” indicating that advanced disease does not reliably predict desired resuscitative interventions, despite the poor outcomes.^23^ It has long been known that patients and their families grossly overestimate the benefit of cardiopulmonary resuscitation.^16^,^25^ Engaging patients with end-stage disease and their families on the realities of resuscitation is an important conversation, and one they are more likely to participate in if they perceive it to be personally relevant.^16^ Further, patients with end-stage disease who participate in end-of-life care discussions with their caregivers and healthcare providers experience much lower rates of mechanical ventilation, ICU admission, and invasive resuscitation.^26^,^27^

Consistent with previous studies, place of residence was strongly associated with patients’ likelihood of having accessible code status documentation.^18^,^28^ Three in four of our recruited patients lived at home, emphasizing the need to target these patients regarding code status discussions.

## LIMITATIONS

This cross-sectional, single-center study was performed at a time when EHRs are rapidly evolving. Despite the small sample size reducing the precision of our estimates, we believe that the broader issue of difficulties in retrieving or demonstrating the absence of code status documentation apply to many healthcare systems and information networks. With respect to the recruitment process, due to staffing restraints our recruitment times for this study omitted the hours 2 am – 6 am, a time when EDs typically have the lowest amount of coverage. While it would have been preferable to recruit during all times of day, because the recruiter was separate from the patients’ care team this four-hour gap in recruitment time should not have significantly impacted our study’s results.

Unfortunately, less than half of patients who were enrolled in the study consented for an interview. The reasons for this are multifactorial: patients declined to an interview because this was their preference; patients lacked the capacity to consent for an interview and an SDM was not available; or they were so critically ill that it was inappropriate to consent a patient (or SDM) for an interview. We acknowledge that this limits the robustness of data gleaned from the patient interviews and introduces some element of selection bias. Additionally, the use of dementia as an inclusion criterion was not meant to imply that all such patients are near the end of life. Rather, such patients were included given their exclusion from most prior studies, the challenges of quickly determining capacity, and the progressive and common nature of this disease.

This study took place in an academic, tertiary-care center where typically a member of the care team could be tasked with reviewing a patient’s prior code status documentation. We recognize that not all ED settings have enough physician resources to assign a team member to this task when a patient is in extremis. While the specific deficiencies and solutions may vary, we hope to draw attention to issues including EHR design, validity of advance directives, role of substitute decision makers, and institutional practices surrounding code status, as the importance of documenting and retrieving code status is universal but complex.

## CONCLUSION

Two out of every three patients with end-stage disease presenting to our ED did not have readily accessible code status documentation, possibly placing them at risk of unwanted resuscitation efforts at the end of life. Many of these patients and their families were unaware of this risk. Immediate access to a patient’s code status using an electronic health record should be a quality benchmark of health information systems, and well-designed information platforms hold promise in this regard. Greater efforts are needed to increase the code status documentation rates for patients living with end-stage disease, provided of course that this information is accurate, current, and readily available in the event of an emergency.

## Supplementary Information



## Figures and Tables

**Table 1:** Patient characteristics.

	All patients enrolled (n = 85)	Patients interviewed (n = 32)
Age (median [interquartile range]), years	78 [67–86]	77 [69–87]
Admissions to hospital within the last year (median [interquartile range])	2 [0–4]	1[0–3]
Female	47(55%)	17(53%)
Canadian triage acuity scale		
1	1(1%)	0(0%)
2	20(24%)	7(22%)
3	56(67%)	21(66%)
4	6(7%)	2(6%)
5	0(0%)	0(0%)
Not recorded	2(2%)	2(6%)
Inclusion criteria met		
Palliative care patient or consult	8(9%)	4(13%)
Metastatic malignancy	24(28%)	9(28%)
Chronic obstructive pulmonary disease dependent on home oxygen	20(23%)	10(31%)
Congestive heart failure dependent on home oxygen	6(7%)	0(0%)
Chronic kidney disease on dialysis	13(15%)	6(19%)
Progressive neurodegenerative disease	32(36%)	11(34%)
Arrival to hospital		
Walk-in	21(25%)	17(53%)
Emergency medical services	52(61%)	12(37%)
Transfer	12(14%)	3(9%)
Place of fesidence		
Community	64(75%)	24(75%)
Assisted-living	9(11%)	3(9%)
Long-term care	10(13%)	4(13%)
Not recorded	2(2%)	1(3%)
Presenting complaint		
Dyspnea	16(19%)	7(22%)
General weakness	8(9%)	2(6%)
Consult for another service	7(8%)	2(6%)
Confusion	6(7%)	1(3%)
Fall trauma	5(6%)	2(6%)
Nausea/vomiting	5(6%)	3(9%)
Seizure	4(5%)	0(0%)
Back pain	4(5%)	1(3%)
Other*	36(42%)	14(44%)

* Other: diarrhea, abdominal pain, altered level of consciousness, pressure ulcer, head injury, anxiety, abdominal distension, abnormal lab values, generalized edema, palpitations, fever, gastrointestinal bleed, urinary retention, chest pain, laceration, flank pain, imaging required, hemoptysis, musculoskeletal injury, stroke.

**Table 2:** Code status documentation (N = 85).

	N (%)
Explicit code status documentation obtained	31(36%)
Medium through which code status was available	
Accompanying paper documentation	13(15%)
EHR	28(33%)
Single-click shortcut	20(23%)
Discharge summary	2(2%)
Clinic reports	6(7%)
Patients with a completed “Patient’s Goals of Care Discussion Form” (Appendix B)	13(15%)

Note: In several cases, code status documentation could be obtained through both paper documentation and the EHR. This explains why the sum of paper documentation and EMR documentation exceeds the total cases of explicit code status documentation obtained.

*EHR*, electronic health record.

**Table 3:** Concordance of code status documentation with patient or substitute decision maker report (N = 34).

		Patient (or SDM) stated code status	
		Full code	DNR

Code status on record	Full code	3(9%)	1(3%)**
	DNR	1(3%)*	10(30%)
	Undocumented	9(27%)	10(30%)

Note: There was one instance where the substitute decision maker (SDM) expressed goals of care that were not consistent with the recorded goals of care,* and one instance of two different recorded goals of care.** This accounts for why the total responses in Table 3 is 34 for 32 patients interviewed.

*SDM*, substitute decision maker; *DNR*, do not resuscitate.

**Table 4:** Analysis of factors associated with the presence of goals-of-care documentation.

	Code status documentation		
	Yes	No	OR [95% CI]
Place of residence	8	2	13 [2.5–65]
Long-term care	8	2	13 [2.5–65]
Assisted-living	6	3	6.7 [1.7–26]
Community	14	47	Ref
Gender	14	47	Ref
Male	10	28	0.41 [0.17–1.04]
Female	22	25	Ref
Age by interquartile range			
87–95	9	11	1.7 [0.52–6.6]
77–86	8	15	1.1 [0.34–4.1]
68–76	7	13	1.1 [0.31–4.4]
0–67	7	15	Ref
Number of hospital admissions in last year			
≥3	16	14	2.6 [0.85–8.01]
1–2	8	24	0.76 [0.25–2.5]
0	7	16	Ref
Past intensive care unit admissions			
Yes	7	6	1.7 [0.44–6.2]
No	9	13	Ref
Past invasive resuscitations			
Yes	4	2	3.5 [0.64–21]
No	9	16	Ref

Chi-square analysis performed.

Note: in the above table, “place of residence,” “gender,” “age,” and “number of hospital admissions in last year” were solely from chart review. “Past intensive care unit admissions,” and “past invasive resuscitations” were obtained through a combination of patient interview and chart review.

*OR*, odds ratio; *CI*, confidence interval.
