# Supplementary material for: Code Status Documentation Availability and Accuracy Among Emergency Patients with End-stage Disease
Source: West J Emerg Med. 2021 Apr 27;22(3):628–35. doi: 10.5811/westjem.2020.12.46801 (PMC8203022; doi:10.5811/westjem.2020.12.46801)
Supplement: Supplementary file 1 [file wjem-22-628-s001.docx]

**Appendix A**: Do Not Resuscitate Confirmation (DNR-C) Form in study’s province.


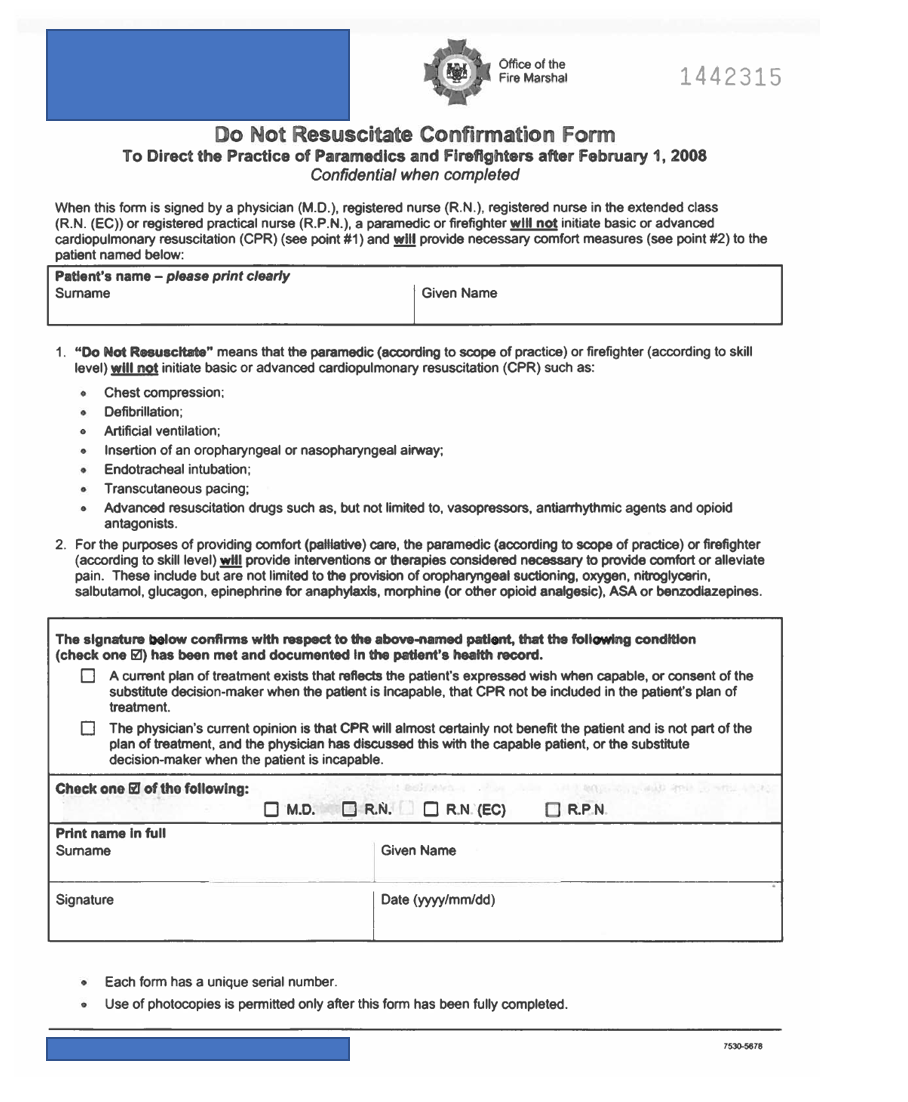


**Appendix B**: Tertiary Care Academic Hospital “Patient’s Goals of Care Discussion Form”


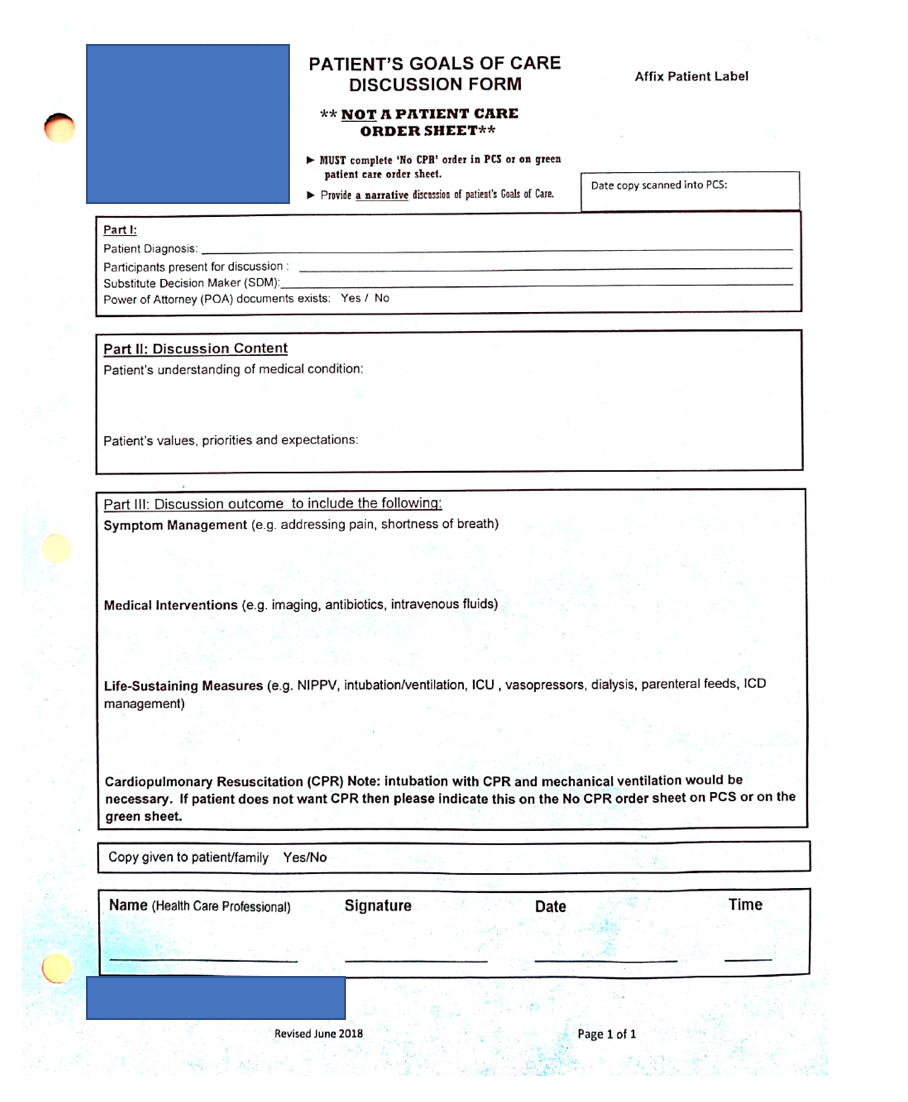


**Appendix C:** Patient interview data (N=32)

|  | N (%)  Combined (n=32) | N (%) Patient  (n=26) | N (%)  SDM  (n=6) |
| --- | --- | --- | --- |
| “If your heart were to stop, or if you were unable to breathe today, would you want measures up to and including chest compression, electric shocks, and a breathing tube if needed?”  Yes  No | 12 (37%)  20 (63%) | 9 (35%)  17 (65%) | 3 (50%)  3 (50%) |
| “Are you aware that in the event that you are very sick or die and come to the emergency department without a substitute decision maker or code status documentation, doctors will use interventions up to and including chest compressions, electric shocks, and a breathing tube if needed.”  Yes I am aware  No I am not aware | 18 (56%)  14 (44%) | 12 (46%)  14 (54%) | 6 (100%)  0 (0%) |
| “In the event that you are so sick that you cannot communicate, it is important that emergency medical staff know what health care measures you want and *do not* want performed.”  Yes  No  Indifferent | 27 (85%)  2 (6%)  3 (9%) | 21 (81%)  2 (7.7%)  3 (11%) | 6 (100%)  0 (0%)  0 (0%) |
| Patients with advance directive documentation  Patients who brought it with them to hospital | 12 (37%)  1 (3%) | 9 (35%)  0 (0%) | 3 (50%)  1 (17%) |
